# Removal of benzylidene acetal and benzyl ether in carbohydrate derivatives using triethylsilane and Pd/C

**DOI:** 10.3762/bjoc.9.9

**Published:** 2013-01-14

**Authors:** Abhishek Santra, Tamashree Ghosh, Anup Kumar Misra

**Affiliations:** 1 Bose Institute, Division of Molecular Medicine, P-1/12, C.I.T. Scheme VII-M, Kolkata-700054, India; FAX: 91-33-2355 3886

**Keywords:** benzylidene, glycoside, Pd/C, transfer hydrogenation, triethylsilane

## Abstract

Clean deprotection of carbohydrate derivatives containing benzylidene acetals and benzyl ethers was achieved under catalytic transfer hydrogenation conditions by using a combination of triethylsilane and 10% Pd/C in CH_3_OH at room temperature. A variety of carbohydrate diol derivatives were prepared from their benzylidene derivatives in excellent yield.

## Introduction

**Scheme 1:**
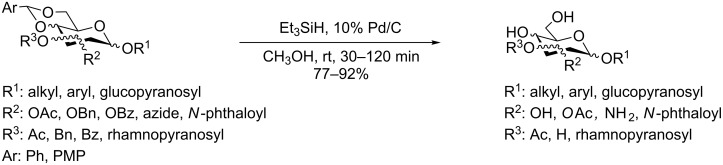
Removal of benzylidene acetal and benzyl ether by using a combination of triethylsilane and 10% Pd/C.

Functionalization of carbohydrate intermediates is essential for their assembly towards the synthesis of complex oligosaccharides [1–9]. Benzylidene acetal is a frequently used protecting group for the simultaneous protection of 1,2- and 1,3-diol derivatives [10–11]. It can be removed under acidic hydrolysis as well as under neutral conditions (e.g., hydrogenolysis). The benzylidene acetal can also be regioselectively opened under reductive conditions to produce partially benzylated derivatives [12–14]. A number of methods have been reported for the removal of benzylidene acetals by using strong protic and Lewis acids [10–11,15] as well as some heterogeneous acidic catalysts [16–17]. Removal of benzylidene acetal under nonacidic conditions includes hydrogenolysis using hydrogen gas over Pd/C [18], or treatment with hydrazine [19] or EtSH [20] or Na/NH_3_ [21], etc. However, most of the above-mentioned methodologies have several shortcomings, such as harsh conditions, formation of unwanted byproducts, longer reaction time, incompatibility of functional groups, use of expensive reagents, fire hazard, etc. In this context, the development of a mild, neutral reaction condition for the removal of benzylidene acetals would be useful in the derivatization of a carbohydrate framework. Mandal et al. reported the removal of benzyl esters/ethers and the reduction of alkenes/alkynes by catalytic transfer hydrogenation using a combination [22] of triethylsilane (Et_3_SiH) and 10% Pd/C. In our synthetic endeavor we sought to explore the catalytic potential of a combination of Et_3_SiH and 10% Pd/C in the deprotection of a benzylidene group in the carbohydrate derivatives, without the formation of unwanted byproducts. We report herein our findings on the removal of benzylidene acetal using a combination of triethylsilane and 10% Pd/C (Scheme 1).

## Results and Discussion

In a set of initial experiments, methyl 2,3-di-*O*-acetyl-4,6-*O*-benzylidene-α-D-glucopyranoside (**1**) was treated with a combination of a varied quantity of triethylsilane (1.0–5.0 equiv of the substrate) and 10% Pd/C (10–20 mg per 100 mg substrate) in CH_3_OH at room temperature. It was observed that the use of a total 3.0 equiv of triethylsilane and 10% Pd/C (10 mg/100 mg of substrate) in CH_3_OH at room temperature furnished the clean removal of the benzylidene group in a 87% yield in 30 min. Generalizing the reaction conditions, a series of benzylidene acetal containing monosaccharide and disaccharide derivatives, with different functional groups present in them, were treated with the optimized reagent system, and clean removal of benzylidene acetal was observed in all cases. The effect of triethylsilane and 10% Pd/C on the removal of benzylidene acetal is presented in Table 1. The noteworthy features of the reaction condition are: (a) neutral conditions; (b) compatibility with a large number of functional groups used in the protection of carbohydrates (e.g., acetyl, benzoyl, phthaloyl, 2-(trimethylsilyl)ethyl, 4-methoxyphenyl, etc.); (c) clean and high yielding; (d) no unwanted byproduct formation; (e) can be carried out at room temperature; (f) no flammable gas is required. The reaction conditions were equally effective for the removal of 4-methoxybenzylidene acetal (Table 1, entries 13–15). Allyl ether was reduced and benzyl ethers were removed under the reaction conditions, as expected from the earlier report [22] (Table 1, entry 8 and entries 3, 4, 9, 10, 12). Benzyl ethers and benzylidene acetal in a compound can be removed in one pot by using these reaction conditions (Table 1, entries 3, 4, 9, 10, 12). All debenzylidenated products were characterized by NMR spectral analysis. Known compounds gave acceptable NMR spectra that matched the previously reported data.

**Table 1:** Conversions of carbohydrate derivatives effected with Et_3_SiH and 10% Pd/C.

Entry	Substrate	Product^a^	Time (min)	Yield^b^ (%)	Ref.^c^

1	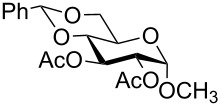 **1**	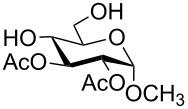 **2**	30	87	[23]
2	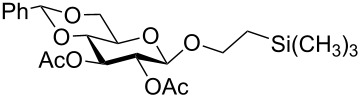 **3**	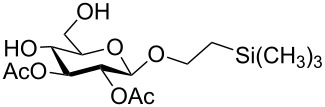 **4**	30	85	[24]
3	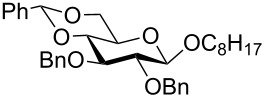 **5**	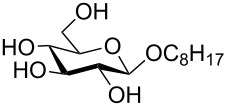 **6**	60	90^d^	[25]
4	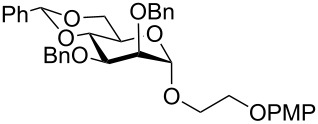 **7**	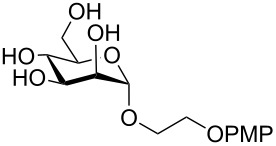 **8**	60	88^d^	–
5	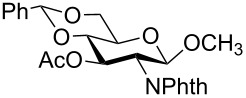 **9**	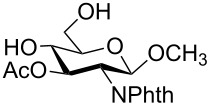 **10**	40	87	[26]
6	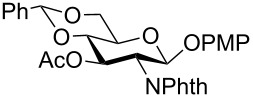 **11**	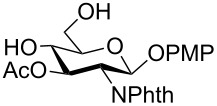 **12**	40	82	[27]
7	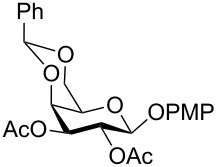 **13**	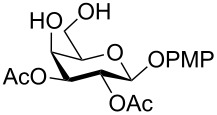 **14**	40	90	–
8	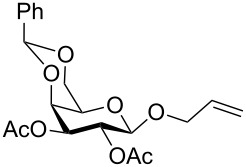 **15**	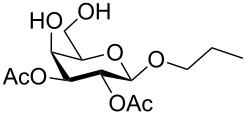 **16**	40	92	–
9	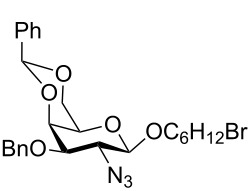 **17**	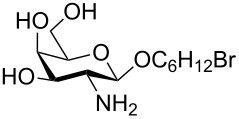 **18**	40	84^d,e^	–
10	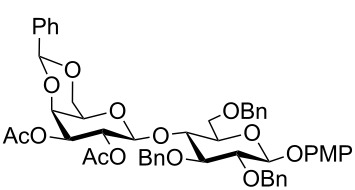 **19**	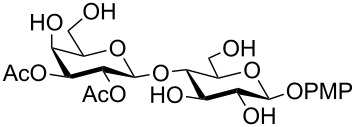 **20**	120	77^d^	[28]
11	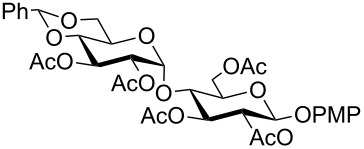 **21**	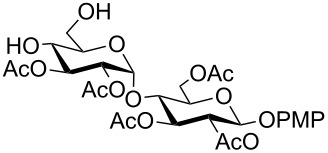 **22**	60	84	–
12	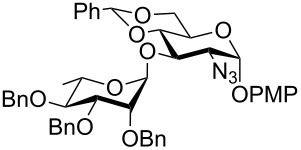 **23**	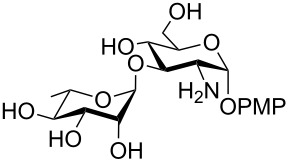 **24**	90	80^d,e^	–
13	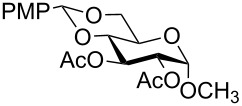 **25**	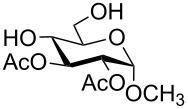 **2**	40	88	[23]
14	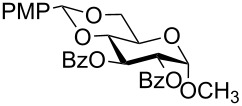 **26**	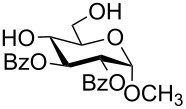 **27**	40	90	[16]
15	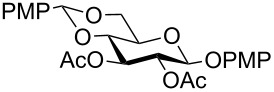 **28**	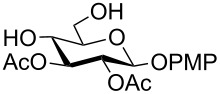 **29**	30	86	[29]

^a^Abbreviations used: PMP: 4-methoxyphenyl; Phth: phthaloyl. ^b^After short-column chromatography; ^c^Preparation reported earlier. ^d^Spectra recorded after per-*O*-acetylation using acetic anhydride and pyridine (1:1; v/v). ^e^Azide group reduced to amine.

## Conclusion

In summary, the efficient deprotection of carbohydrate derivatives containing benzylidene acetal and *O*-benzyl groups under catalytic transfer hydrogenation conditions has been developed by using a combination of triethylsilane and 10% Pd/C. The efficacy of this methodology is comparable to the conventional hydrogenation involving hydrogen gas and Pd/C, whereas it does not require handling of a flammable gas. Being operationally simple and high yielding, these reaction conditions will certainly be accepted as a useful alternative to those currently existing in this area.

## Experimental

### Typical experimental procedure

To a solution of compound **1** (500 mg, 1.36 mmol) and 10% Pd(OH)_2_/C (50 mg) in CH_3_OH (5 mL) was added Et_3_SiH (650 μL, 4.07 mmol) portionwise, and the reaction mixture was stirred at room temperature for an appropriate time, given in Table 1. The reaction mixture was filtered through a Celite bed, and the filtering bed was washed with CH_3_OH. The combined filtrate was concentrated under reduced pressure to give the crude product, which was passed through a short pad of SiO_2_ with EtOAc as eluant to give pure compound **2** (330 mg, 87%).

## Supporting Information

File 1 Analytical data of new compounds, ^1^H NMR and ^13^C NMR spectra.
