# Supplementary material for: Removal of benzylidene acetal and benzyl ether in carbohydrate derivatives using triethylsilane and Pd/C
Source: Beilstein J Org Chem. 2013 Jan 14;9:74–8. doi: 10.3762/bjoc.9.9 (PMC3566760; doi:10.3762/bjoc.9.9)
Supplement: File 1 — Analytical data of new compounds, 1H NMR and 13C NMR spectra. [file Beilstein_J_Org_Chem-09-74-s001.pdf]

Supporting Information

for

Removal of benzylidene acetal and benzyl ether in carbohydrate derivatives using triethylsilane and Pd/C

Abhishek Santra, Tamashree Ghosh and Anup Kumar Misra*

Address: Bose Institute, Division of Molecular Medicine, P-1/12, C.I.T. Scheme VII-M, Kolkata-700054, India; FAX: 91-33-2355 3886

Email: Anup Kumar Misra - akmisra69@gmail.com

*Corresponding author

Analytical data of new compounds, ^{1}H NMR and ^{13}C NMR spectra

Analytical data of new compounds: acetylated-**8**, **14**, **16**, acetylated-**18**, **22** and acetylated-**24** S2

NMR spectra of compounds **2**, **4**, acetylated-**6**, acetylated-**8**, **10**, **12**, **14**, **16**, acetylated-**18**, **22**, acetylated-**24**, **27**, **29** S5

Analytical data of new compounds

2-(*p*-Methoxyphenoxy)ethyl 2,3,4,6-tetra-*O*-acetyl-α-D-mannopyranoside (acetylated-8): Colorless oil; $[\alpha]_D^{25}$ + 39 (*c* 1.0, $CHCl_3$); IR (neat): 2566, 1759, 1377, 1229, 1166, 1053, 916 cm^{-1}; ^1H NMR ($CDCl_3$, 500 MHz): δ 6.78-6.73 (m, 4 H, Ar-H), 5.24 (dd, *J* = 10.0 Hz, 3.0 Hz, 1 H, H-3), 5.20-5.19 (m, 1 H, H-2), 5.18 (t, *J* = 10.0 Hz each, 1 H, H-4), 4.83 (d, *J* = 1.5 Hz, 1 H, H-1), 4.19 (dd, *J* = 12.0 Hz, 5.0 Hz, 1 H, H-6_a), 4.08-4.04 (m, 2 H, H-5, H-6_b), 4.01-3.96 (m, 2 H, CH_2), 3.94-3.90 (m, 1 H, CH_2), 3.83-3.78 (m, 1 H, CH_2), 3.69 (s, 3 H, OCH_3), 2.09, 2.01, 1.96, 1.92 (4s, 12 H, 4 $COCH_3$); ^{13}C NMR ($CDCl_3$, 125 MHz): δ 170.4, 169.8, 169.6, 169.5 (4 $COCH_3$), 154.2-114.7 (Ar-C), 97.7 (C-1), 69.5 (C-2), 69.0 (C-3), 68.6 (OCH_2), 67.4 (OCH_2), 66.9 (C-4), 66.1 (C-5), 62.3 (C-6), 55.6 (OCH_3), 20.9, 20.7, 20.6 (2 C) (4 $COCH_3$); ESIMS: 521.1 $[M + Na]^+$; Anal. calcd for $C_{23}H_{30}O_{12}$ (498.17): C, 55.42; H, 6.07; found: C, 55.25; H, 6.26.

***p*-Methoxyphenyl 2,3-di-*O*-acetyl-β-D-galactopyranoside (14)**: Colorless oil; $[\alpha]_D^{25}$ + 14 (*c* 1.0, $CHCl_3$); IR (neat): 2988, 2942, 1748, 1433, 1377, 1226, 1085, 1056, 918, 756 cm^{-1}; ^1H NMR ($CDCl_3$, 500 MHz): δ 6.84 (d, *J* = 9.0 Hz, 2 H, Ar-H), 6.70 (d, *J* = 9.0 Hz, 2 H, Ar-H), 5.40 (dd, *J* = 8.0 Hz each, 1 H, H-2), 4.90 (dd, *J* = 10.5, 3.5 Hz, 1 H, H-3), 4.85 (d, *J* = 8.0 Hz, 1 H, H-1), 4.11 (br s, 1 H, H-4), 3.84-3.80 (m, 2 H, H-6_{ab}), 3.67 (s, 3 H, OCH_3), 3.62- 3.60 (m, 1 H, H-5), 3.44, 2.92 (2 br s, 1 H each, 2 O*H*), 2.03, 1.99 (2s, 6 H, 2 $COCH_3$); ^{13}C NMR ($CDCl_3$, 125 MHz): δ 170.3, 169.6 (2 $COCH_3$), 155.5-114.6 (Ar-C), 100.7 (C-1), 74.4 (C-5), 73.4 (C-3), 69.3 (C-4), 67.8 (C-2), 62.0 (C-6), 55.5 (OCH_3), 20.8, 20.7 (2 $COCH_3$); ESIMS: 393.1 $[M + Na]^+$; Anal. calcd for $C_{17}H_{22}O_9$ (370.12): C, 55.13; H, 5.99; found: C, 54.97; H, 6.25.

***n*-Propyl 2,3-di-*O*-acetyl-β-D-galactopyranoside (16)**: Colorless oil; $[\alpha]_D^{25}$ + 2 1.0, $CHCl_3$); IR (neat): 2928, 2853, 1744, 1371, 1222, 1045, 914, 761 cm^{-1}; ^1H NMR ($CDCl_3$, 500 MHz): δ 5.17 (dd, *J* = 8.0 Hz each, 1 H, H-2), 4.83 (dd, *J* = 10.5, 3.5 Hz, 1 H, H-3), 4.39 (d, *J* = 7.5 Hz, 1 H, H-1), 4.08 (d, *J* = 3.0 Hz, 1 H, H-4), 3.85-3.76 (m, 3 H, H-6_{ab}, CH_2), 3.53-3.51 (m, 1 H, H-5), 3.38-3.34 (m, 1 H, CH_2), 2.02, 1.97 (2 s, 6 H, 2 $COCH_3$), 1.55-1.45 (m, 2 H, CH_2), 0.85 (t, *J* = 7.5 Hz each, 3 H, CH_3); ^{13}C NMR ($CDCl_3$, 125 MHz): δ 170.3, 169.6 (2 $COCH_3$), 101.3 (C-1), 74.0 (C-5), 73.5 (OCH_2), 71.6 (C-3), 69.5 (C-2), 68.0 (C-4), 62.0 (C-6), 22.7 (CH_2), 20.8, 20.7 (2 $COCH_3$), 10.3 (CH_3); ESIMS: 329.1 $[M + Na]^+$; Anal. calcd for $C_{13}H_{22}O_8$ (306.13): C, 50.97; H, 7.24; found: C, 50.79; H, 7.46.

6-Bromohexyl 2-acetamido-3,4,6-tri-*O*-acetyl-2-deoxy-α-D-galactopyranoside (acetylated-18): Colorless oil; $[\alpha]_D^{25}$ + 55 (*c* 1.0, $CHCl_3$); IR (neat): 2994, 2947, 1756, 1444, 1356, 1233, 1087, 1056, 915, 756 cm^{-1}; ^{1}H NMR ($CDCl_3$, 500 MHz): δ 5.59 (d, *J* = 9.5 Hz, 1 H, N*H*), 5.35 (d, *J* = 3.0 Hz, 1 H, H-1), 5.15 (dd, *J* = 11.0, 3.0 Hz, 1 H, H-3), 4.85 (d, *J* = 4.0 Hz, 1 H, H-4), 4.56- 4.51 (m, 1 H, H-5), 4.13- 4.05 (m, 3 H, H-2, H-6_{ab}),3.71-3.66 (m, 1 H, OCH_2), 3.47-3.42 (m, 1 H, OCH_2), 3.32-3.26 (m, 2 H, CH_2), 2.17, 2.04, 2.00, 1.96 (4 s, 12 H, 4 COCH_3), 1.64-1.61 (m, 4 H, CH_2); ^{13}C NMR ($CDCl_3$, 125 MHz): δ 170.8, 170.2 (2 C), 169.7 (4 *C*OCH_3), 97.6 (C-1), 68.4 (C-3), 68.2 (O*C*H_2), 67.3 (C-5), 66.6 (C-4), 61.8 (C-6), 51.2 (C-2), 47.9 (*C*H_2), 29.2 (*C*H_2), 28.8 (*C*H_2), 26.5 (*C*H_2), 25.8 (*C*H_2), 23.2 (NHCO*C*H_3), 20.7 (2 C), 20.6 (3 CO*C*H_3); ESIMS: 532.1 $[M + Na]^+$; Anal. calcd for $C_{20}H_{32}BrNO_9$ (509.12): C, 47.07; H, 6.32; found: C, 46.90; H, 6.46.

***p*-Methoxyphenyl *O*-(2,3-di-*O*-acetyl-α-D-glucopyranosyl)-(1→4)-2,3,6-tri-*O*-acetyl-β-D-glucopyranoside (22)**: Colorless oil; $[\alpha]_D^{25}$ + 43 (*c* 1.0, $CHCl_3$); IR (neat): 2928, 2856, 1746, 1433, 1372, 1229, 1041, 759, 602 cm^{-1}; ^{1}H NMR ($CDCl_3$, 500 MHz): δ 6.90 (d, *J* = 9.0 Hz, 2 H, Ar-H), 6.76 (d, *J* = 9.0 Hz, 2 H, Ar-H), 5.33 (d, *J* = 3.5 Hz, 1 H, H-1_B), 5.27 (t, *J* = 9.0 Hz each, 1 H, H-2_A), 5.19 (t, *J* = 9.0 Hz each, 1 H, H-3_A), 5.03 (t, *J* = 8.0 Hz each, 1 H, H-3_B), 4.97 (t, *J* = 8.0 Hz, 1 H, H-1_A), 4.71 (dd, *J* = 10.5, 3.0 Hz, 1 H, H-2_B), 4.50 (d, *J* = 12.0 Hz, 1 H, H-6_{aA}), 4.21 (dd, *J* = 12.0, 5.0 Hz, 1 H, H-6_{bA}), 4.05 (t, *J* = 9.5 Hz each, 1 H, H-4_B), 3.82-3.76 (m, 3 H, H-4_A, H-5_A, H-5_B), 3.74 (s, 3 H, OCH_3), 3.63-3.59 (m, 2 H, H-6_{abB}), 2.09, 2.06, 2.02, 2.00 (5 s, 15 H, 5 COCH_3); ^{13}C NMR ($CDCl_3$, 125 MHz): δ 171.1, 171.8, 170.7, 170.2, 169.5 (5 *C*OCH_3), 155.7-114.5 (Ar-C), 99.5 (C-1_B), 95.7 (C-1_A), 75.4 (C-3_B), 72.8 (C-3_A), 72.3 (C-4_A), 72.2 (C-4_B), 72.0 (C-2_B), 71.8 (C-2_A), 70.4 (C-5_B), 69.2 (C-5_A), 62.9 (C-6_B), 61.8 (C-6_A), 55.5 (O*C*H_3), 20.9, 20.8 (2 C), 20.6, 20.5 (5 CO*C*H_3); ESIMS: 681.2 $[M + Na]^+$; Anal. calcd for $C_{29}H_{38}O_{17}$ (658.21): C, 52.89; H, 5.82; found: C, 52.74; H, 6.00.

***p*-Methoxyphenyl *O*-(2,3,4-tri-*O*-acetyl-α-L-rhamnopyranosyl)-(1→3)-2-acetamido-4,6-di-*O*-acetyl-2-deoxy-α-D-glucopyranoside (acetylated-24):** Colorless oil; $[\alpha]_D^{25}$ + 63 (*c* 1.0, $CHCl_3$); IR (neat): 2957, 2924, 2592, 1745, 1433, 1376, 1239, 1044, 890 cm^{-1}; ^{1}H NMR ($CDCl_3$, 500 MHz): δ 6.98 (d, *J* = 9.0 Hz, 2 H, Ar-H), 6.83 (d, *J* = 9.0 Hz, 2 H, Ar-H), 5.86 (dd, *J* = 9.5, 3.5 Hz, 1 H, N*H*), 5.41 (d, *J* = 3.5 Hz, 1 H, H-1_A), 5.17-5.11 (m, 3 H, H-2_B, H-3_B, H-4_B), 5.04 (t, *J* = 10.0 Hz each, 1 H, H-4_A), 4.86 (br s, 1 H, H-1_B), 4.57 (t, *J* = 10.5 Hz each, 1 H, H-2_A), 4.15-4.09 (m, 2 H, H-6_{abA}), 4.03-3.90 (m, 3 H, H-3_A, H-5_A, H-5_B), 3.77 (s, 3 H, OCH_3), 2.16, 2.11, 2.08, 2.04, 2.03, 1.97 (6 COCH_3), 1.14 (d, *J* = 6.0 Hz, 3 H, CCH_3); ^{13}C NMR ($CDCl_3$,

125 MHz): δ 170.5 (4 C), 169.5 (2 C) (6 *C*OCH$_3$), 155.5-114.8 (Ar-C), 99.8 (C-1$_A$), 97.0 (C-1$_B$), 80.3 (C-3$_A$), 70.5 (C-5$_A$), 70.1 (C-2$_B$), 69.9 (C-3$_B$), 68.9 (C-4$_A$), 68.6 (C-4$_B$), 67.6 (C-5$_B$), 61.8 (C-6$_A$), 55.6 (O*C*H$_3$), 51.9 (C-2$_A$), 23.2 (NHCO*C*H$_3$), 21.1, 21.0, 20.8, 20.7, 20.6 (5 CO*C*H$_3$), 17.2 (C*C*H$_3$); ESIMS: 706.2 $[M + Na]^+$; Anal. calcd for $C_{31}H_{41}NO_{16}$ (683.24): C, 54.46; H, 6.04; found: C, 54.30; H, 6.22.

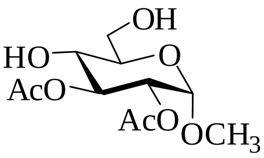

2

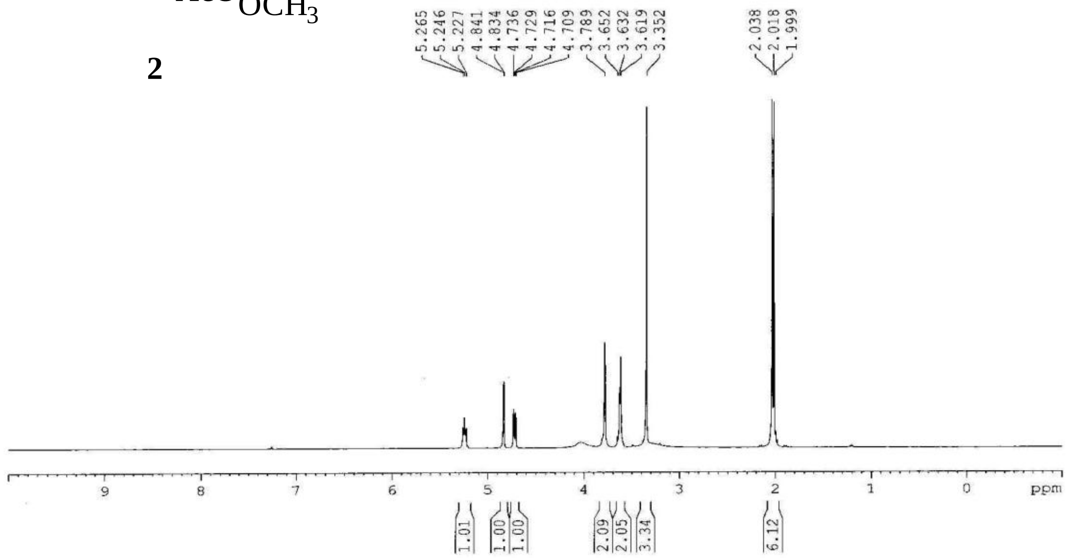

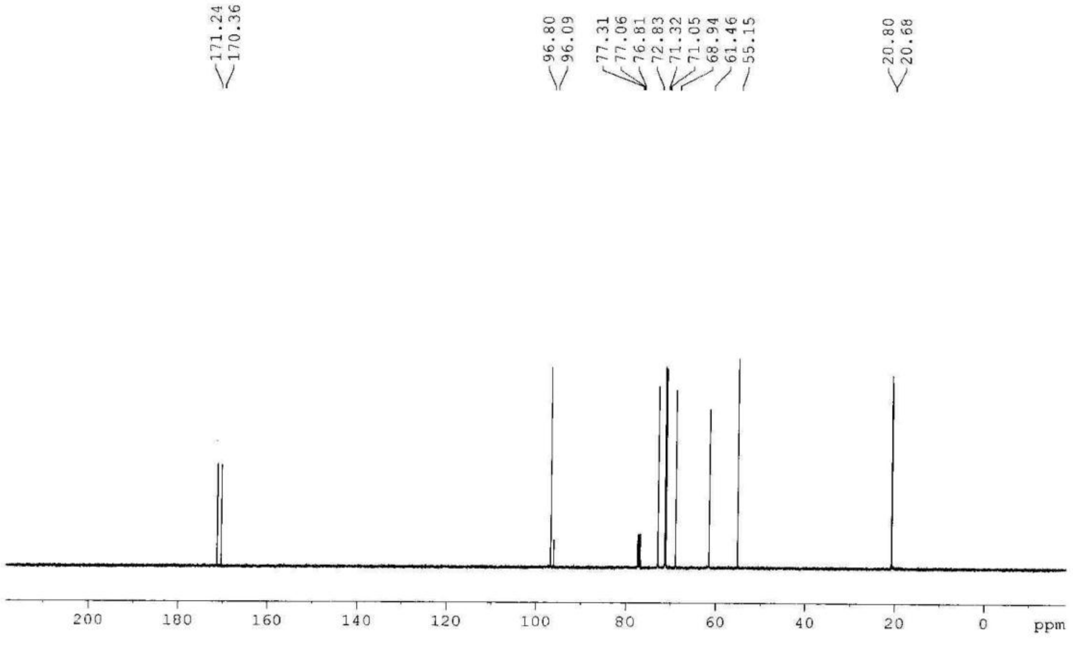

^{1}H NMR (500 MHz) and ^{13}C NMR (125 MHz) spectra in $CDCl_3$.

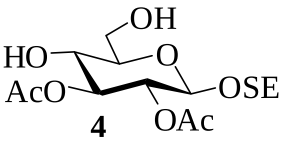

4

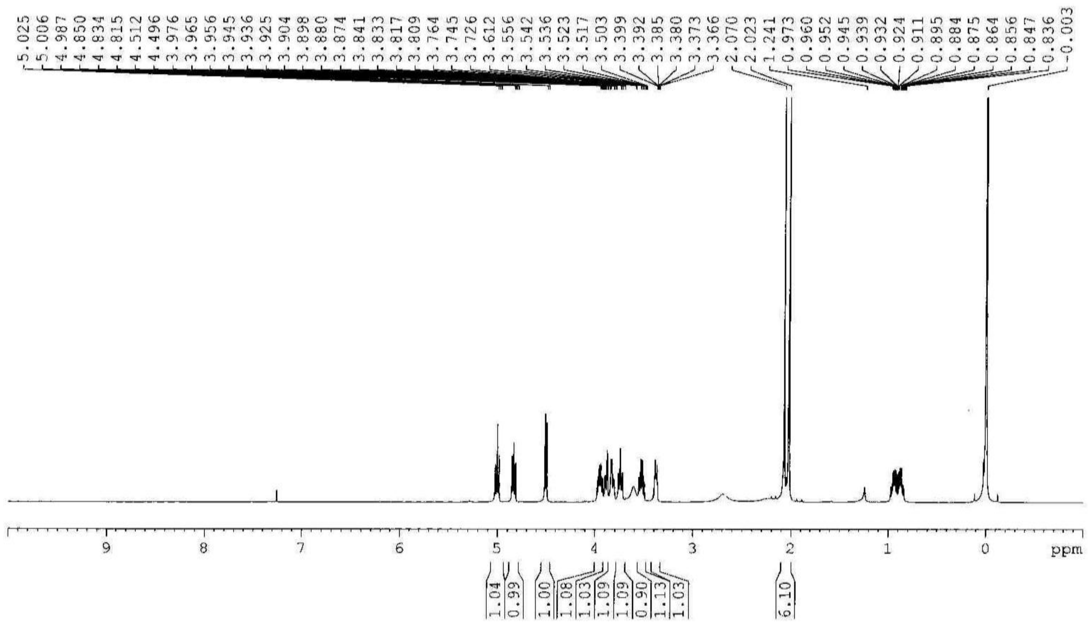

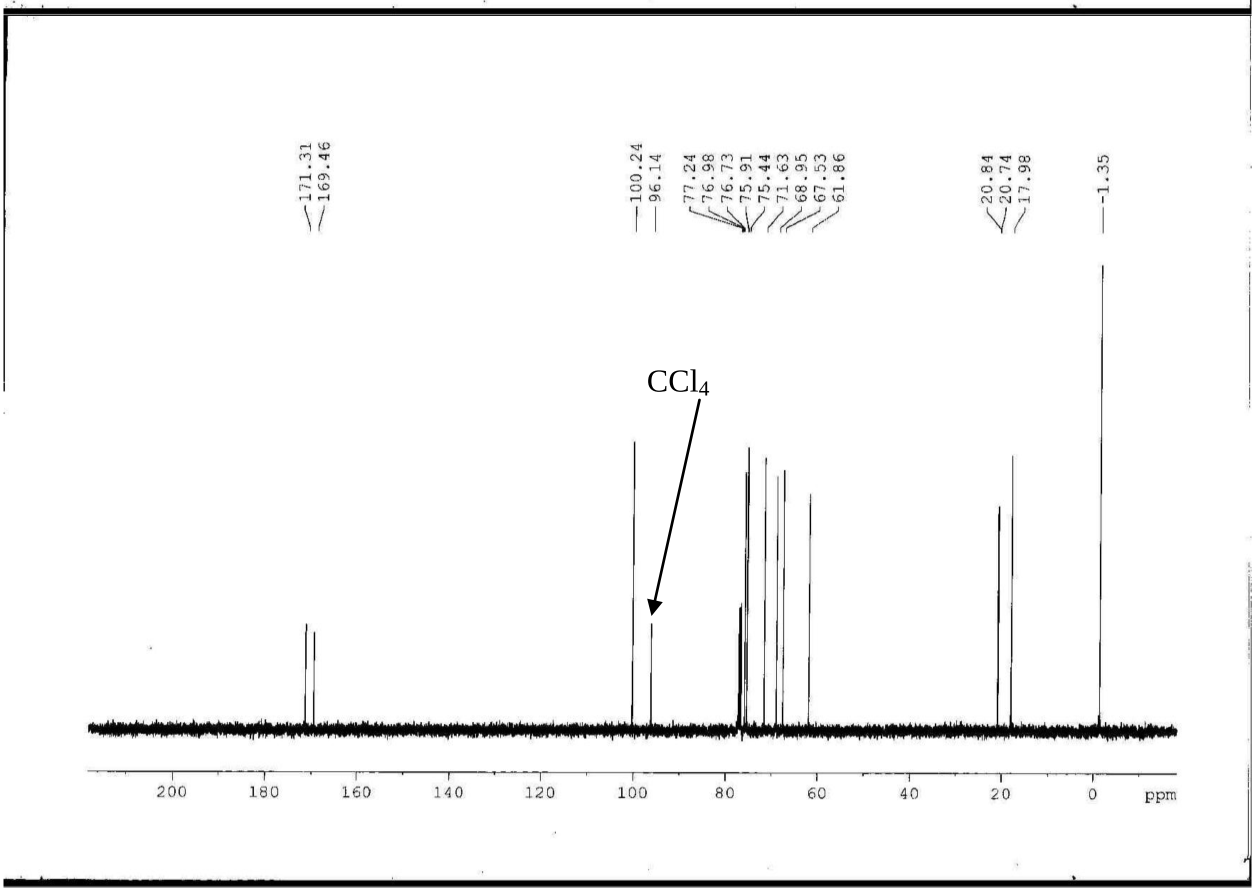

^{1}H NMR (500 MHz) and ^{13}C NMR (125 MHz) spectra in $CDCl_3$.

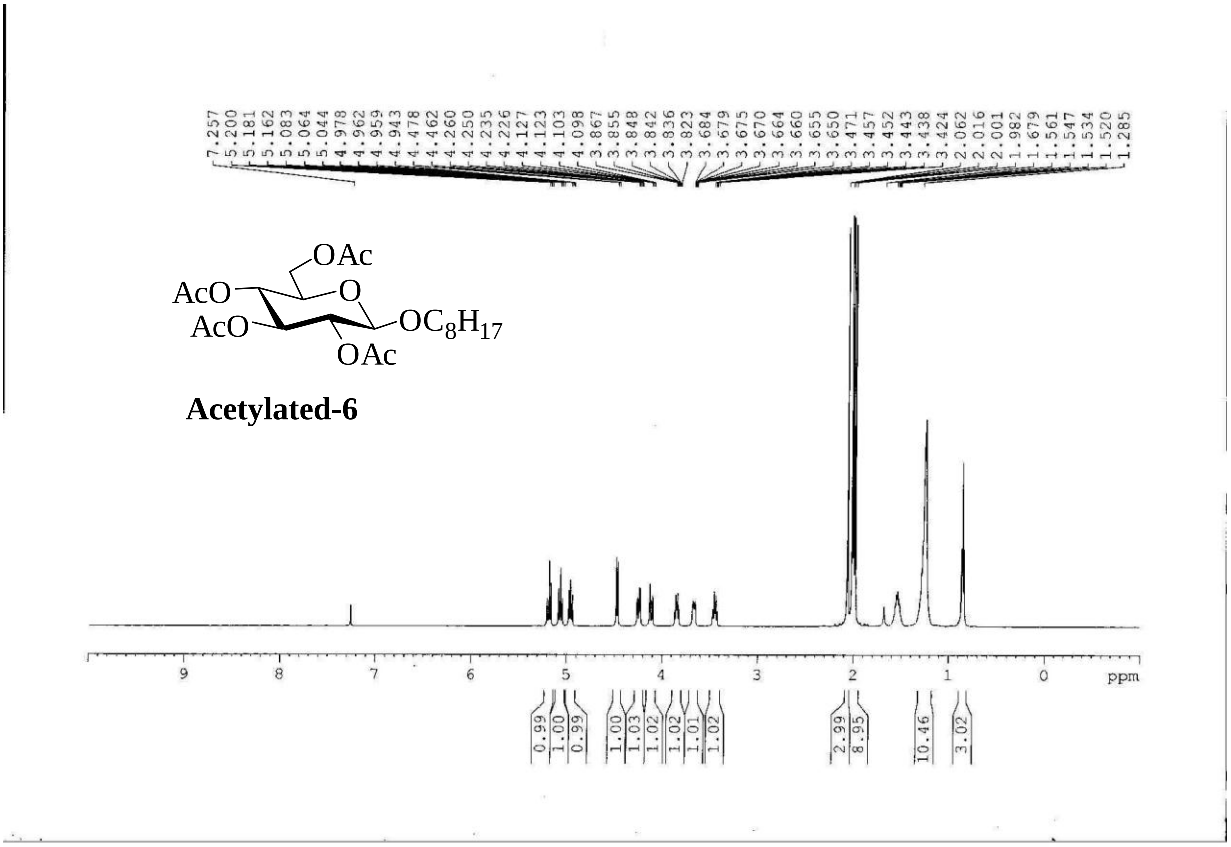

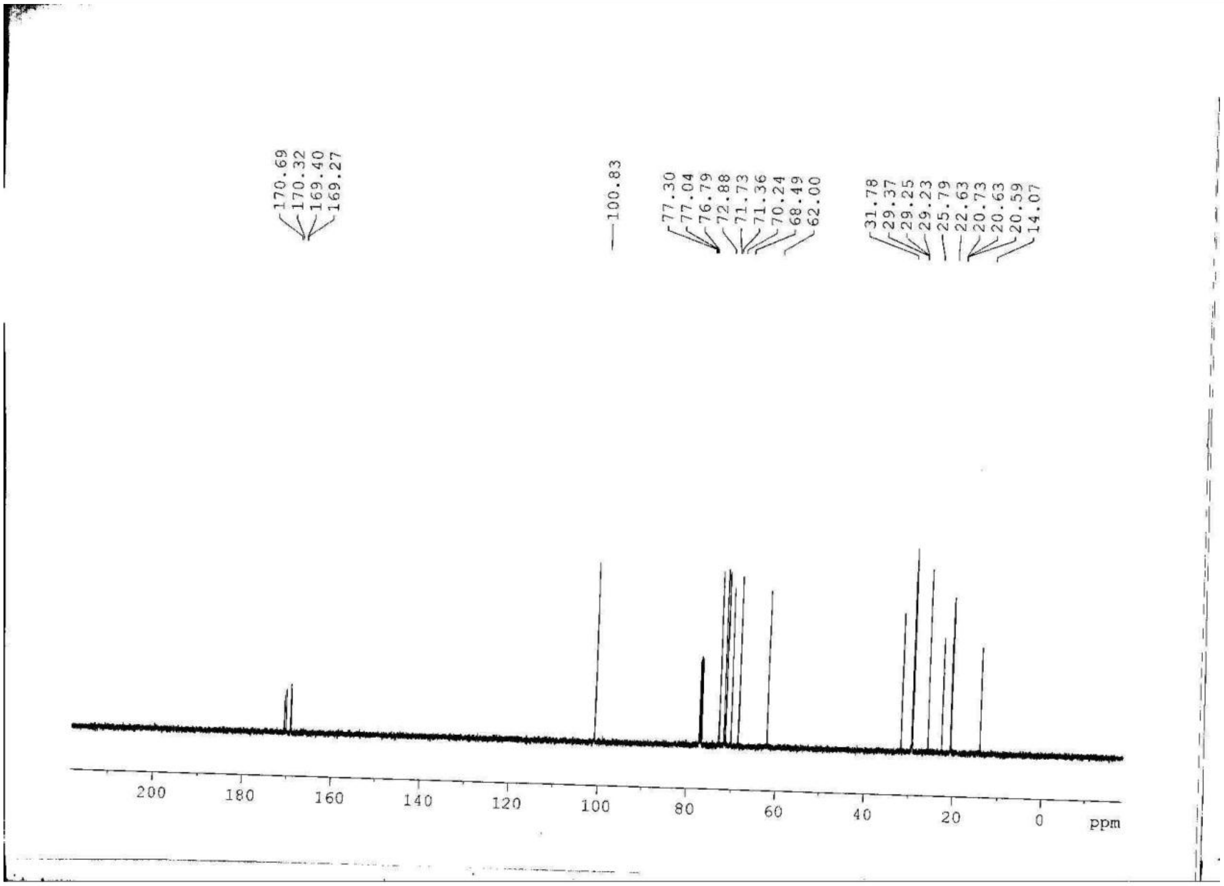

^{1}H NMR (500 MHz) and ^{13}C NMR (125 MHz) spectra in $CDCl_3$.

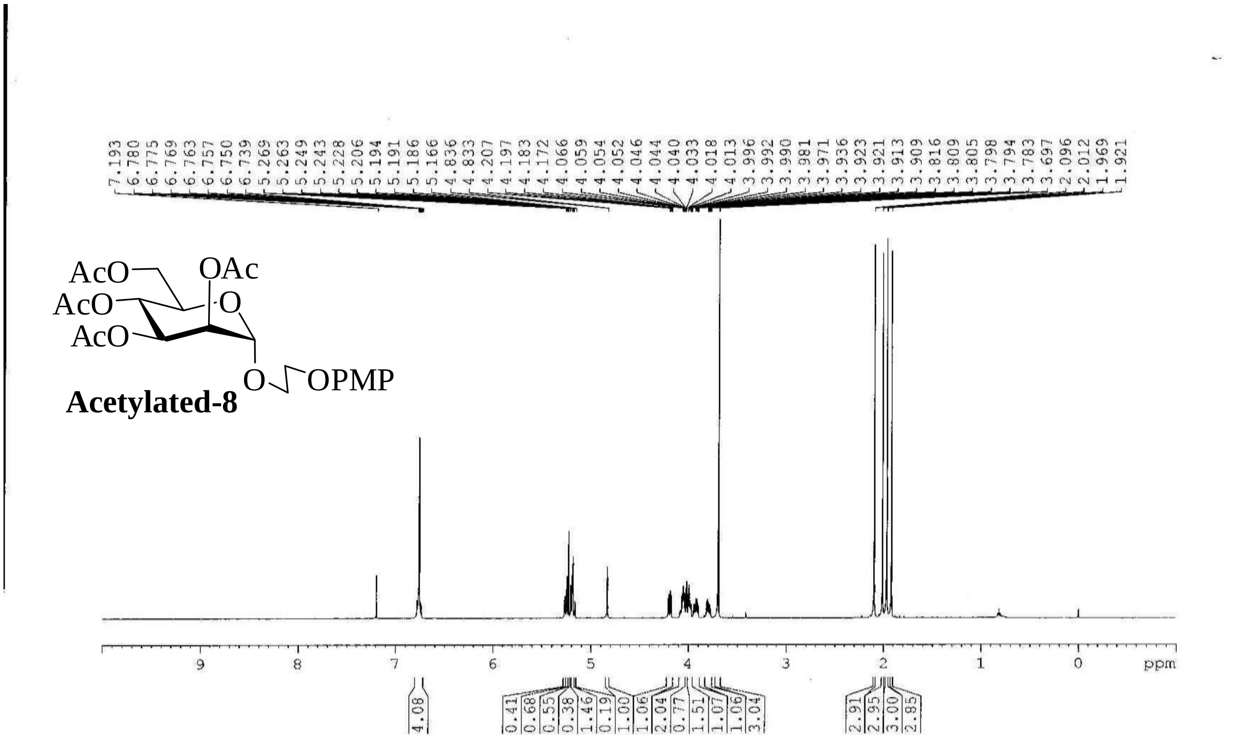

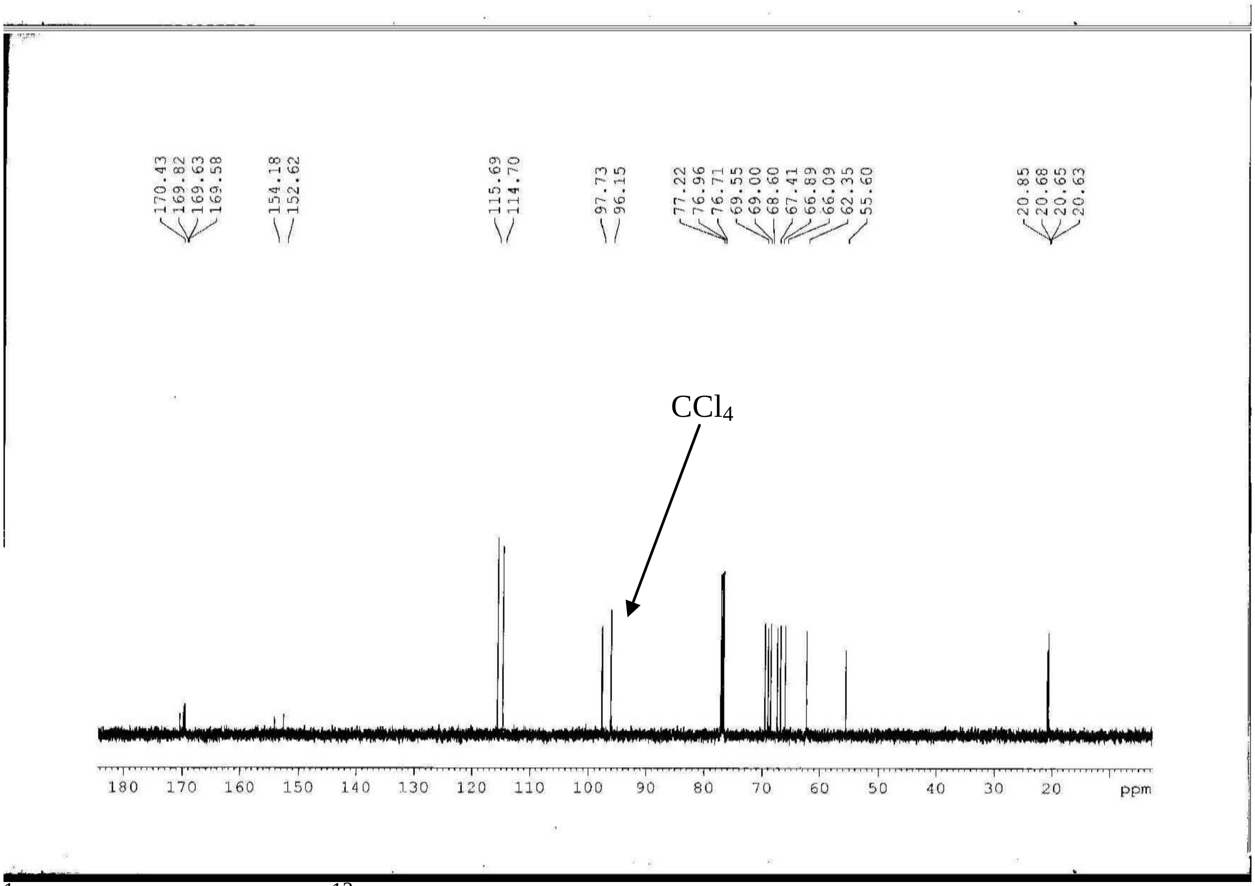

^{1}H NMR (500 MHz) and ^{13}C NMR (125 MHz) spectra in $CDCl_3$.

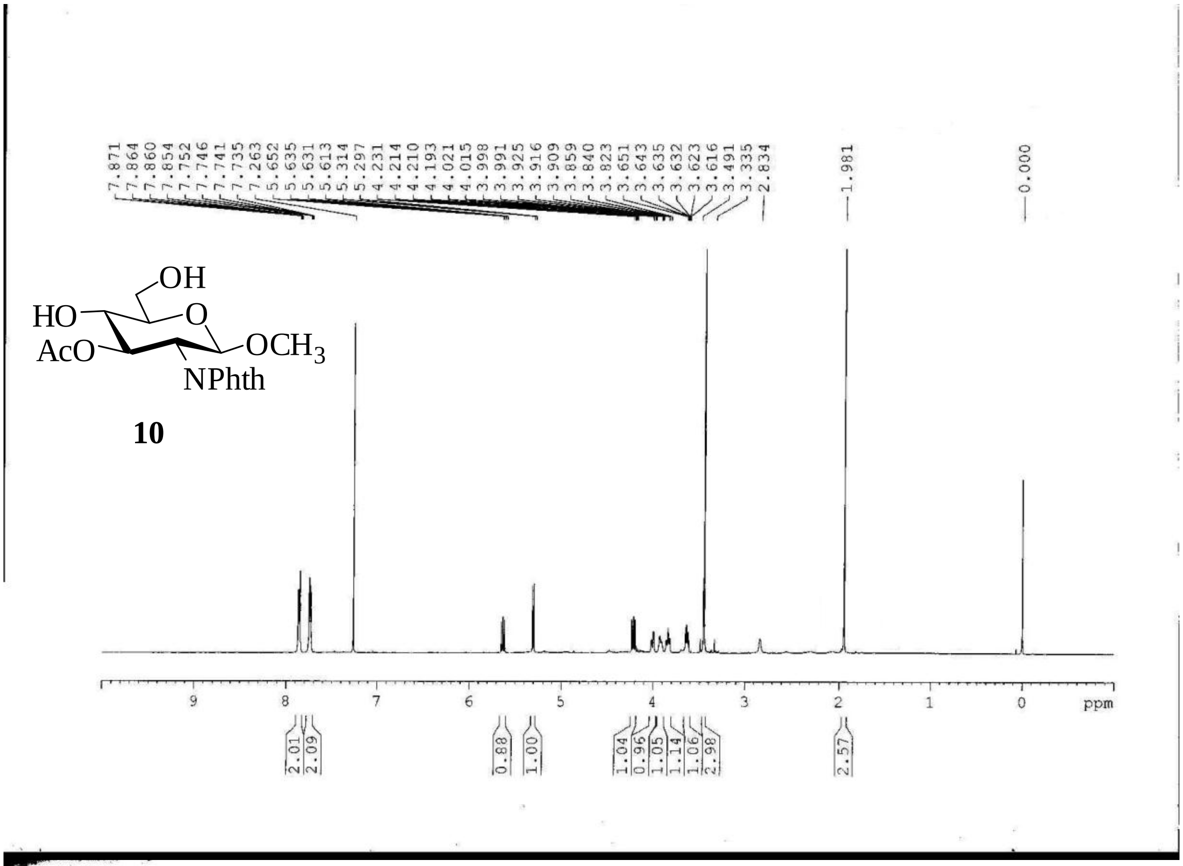

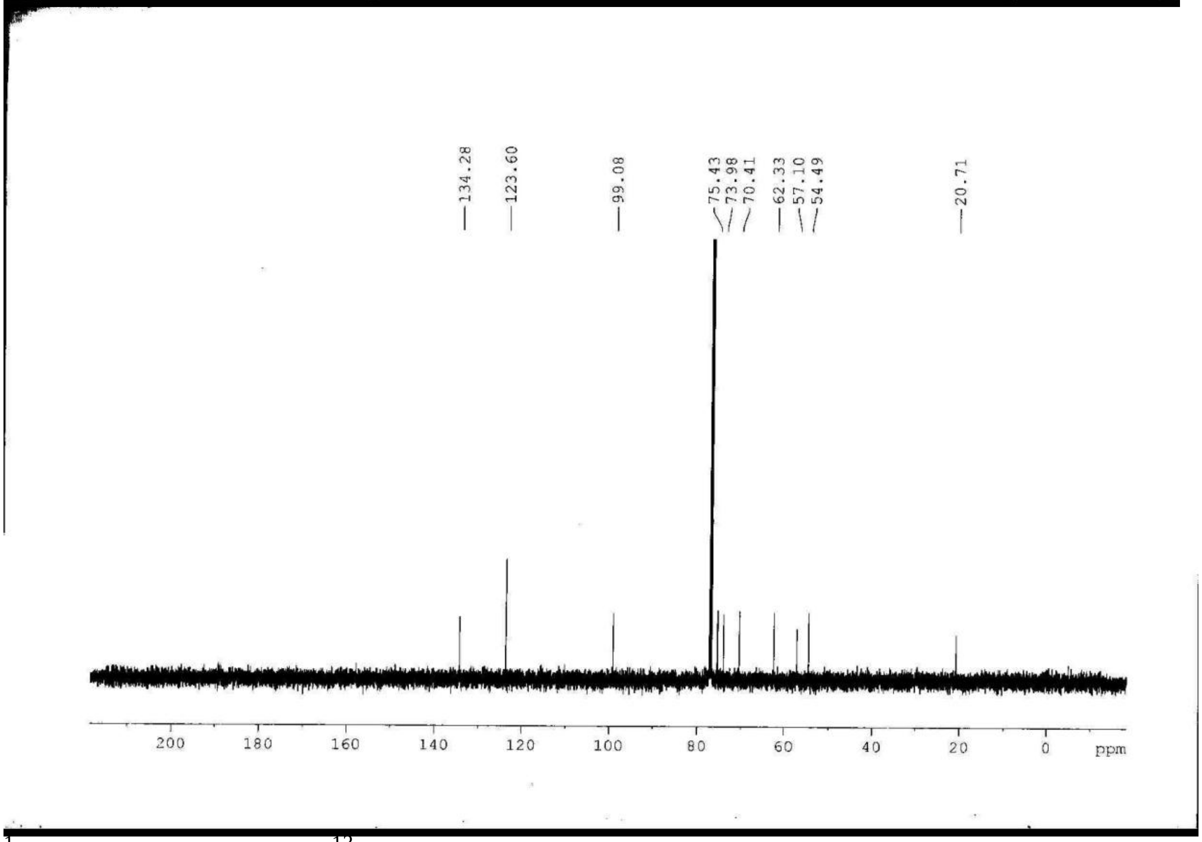

^{1}H NMR (500 MHz) and ^{13}C NMR (125 MHz) spectra in $CDCl_3$.

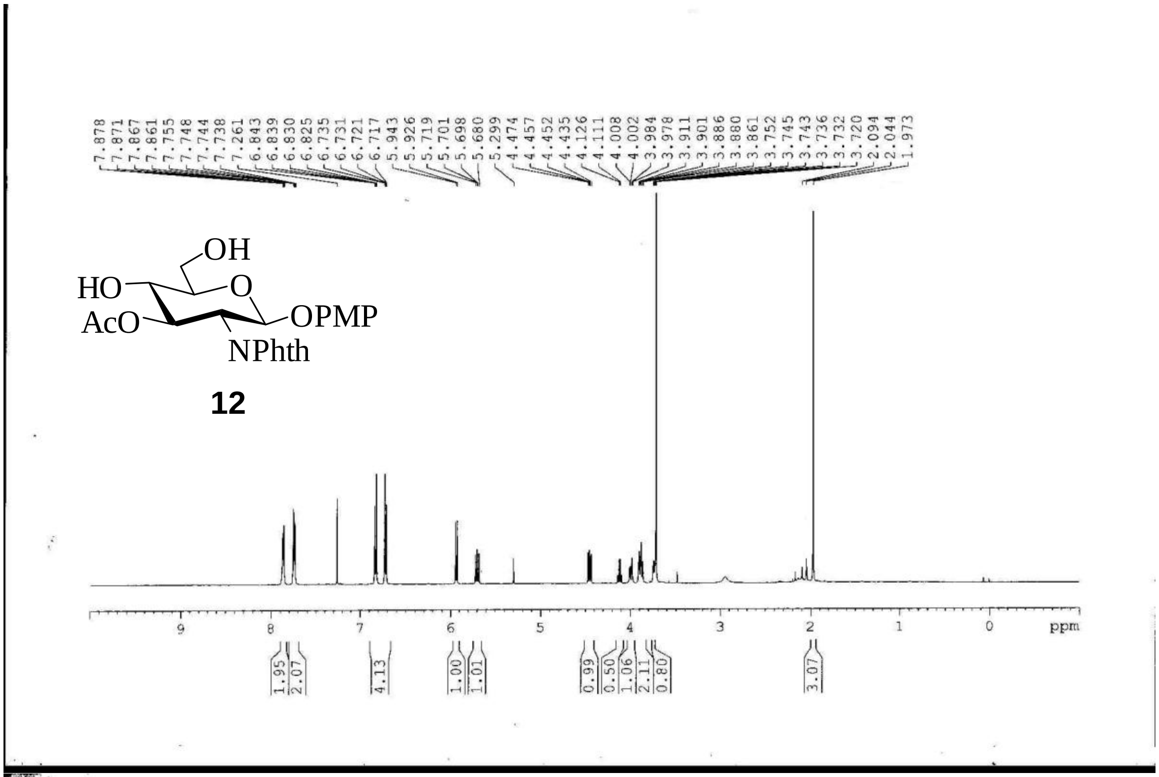

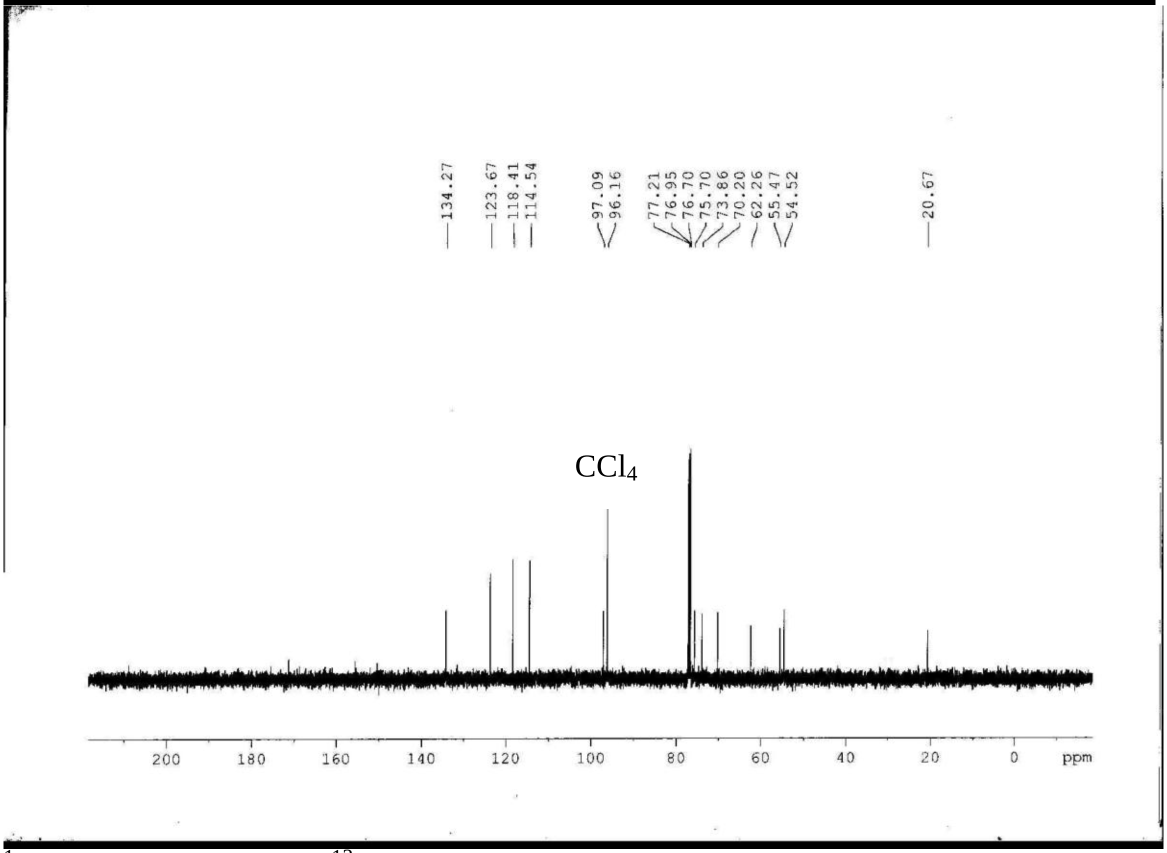

^{1}H NMR (500 MHz) and ^{13}C NMR (125 MHz) spectra in $CDCl_3$.

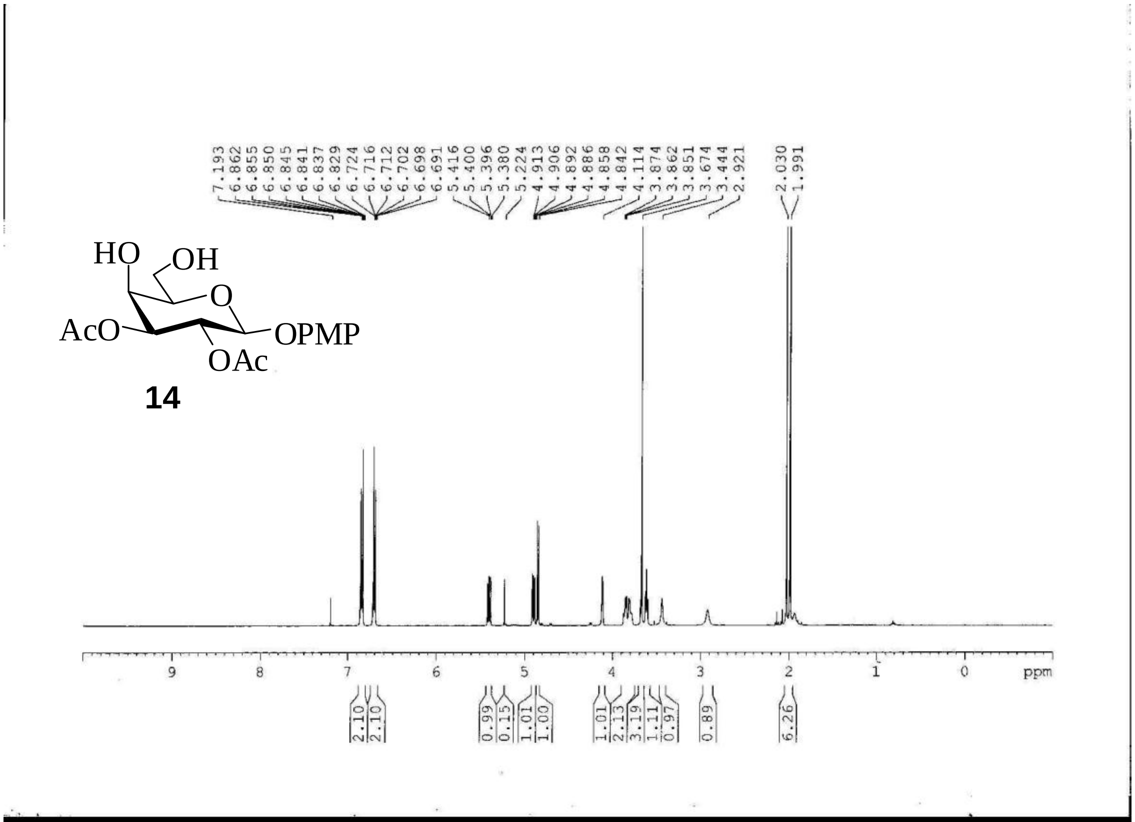

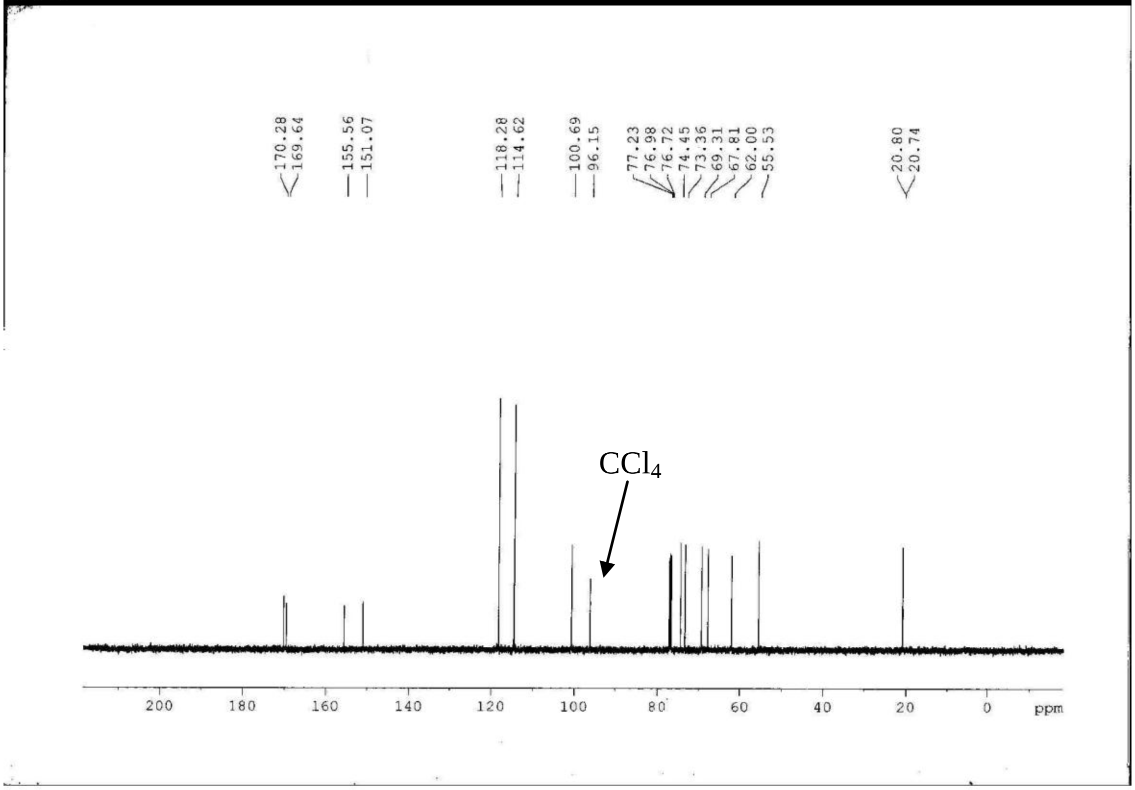

^{1}H NMR (500 MHz) and ^{13}C NMR (125 MHz) spectra in $CDCl_3$.

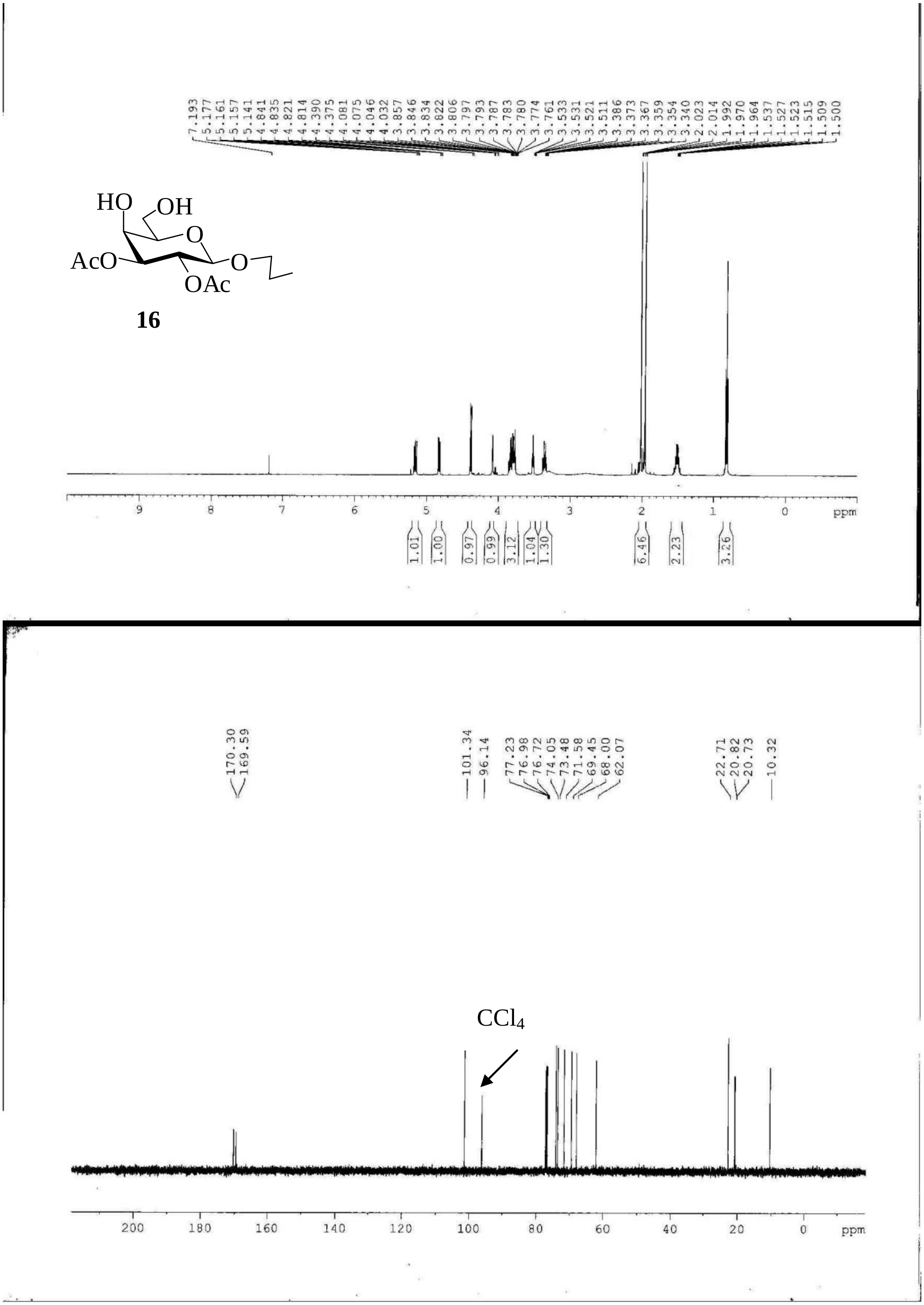

^{1}H NMR (500 MHz) and ^{13}C NMR (125 MHz) spectra in $CDCl_3$.

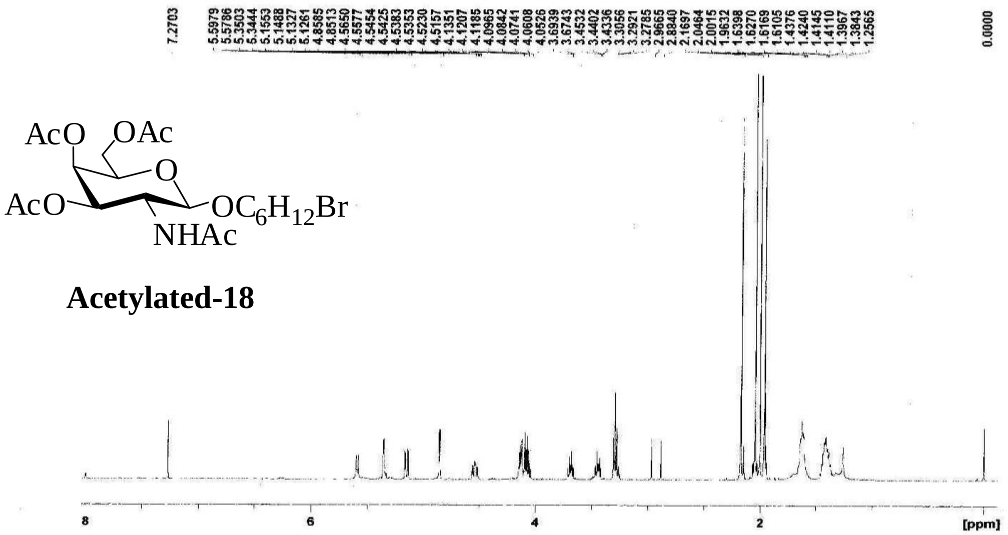

Acetylated-18

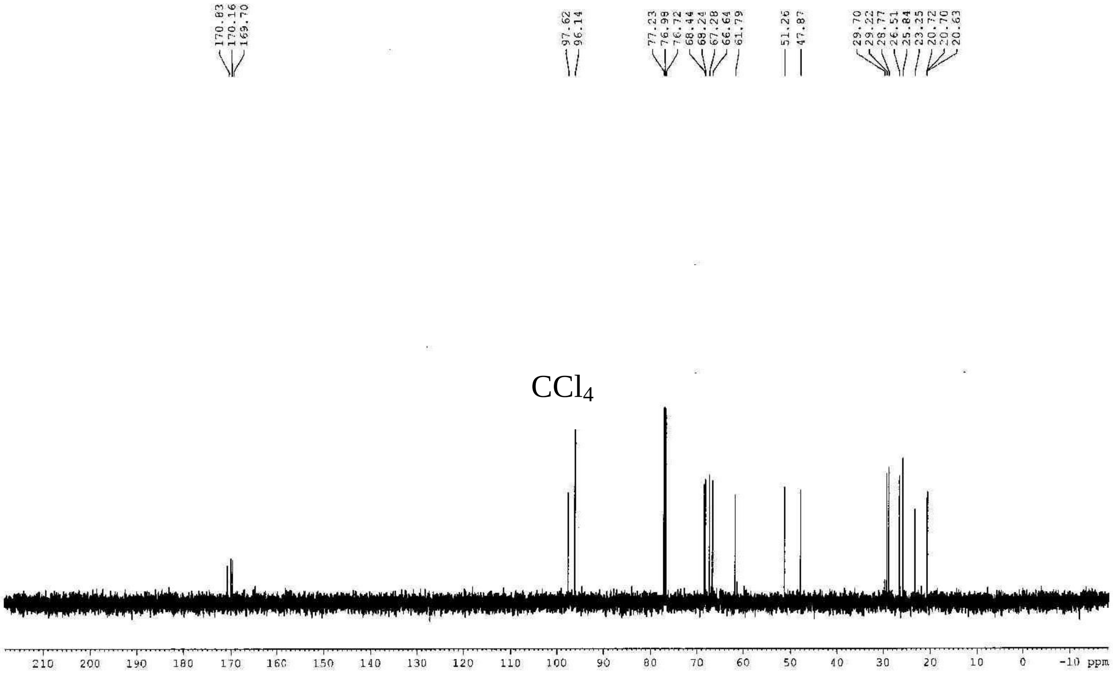

^{1}H NMR (500 MHz) and ^{13}C NMR (125 MHz) spectra in $CDCl_3$.

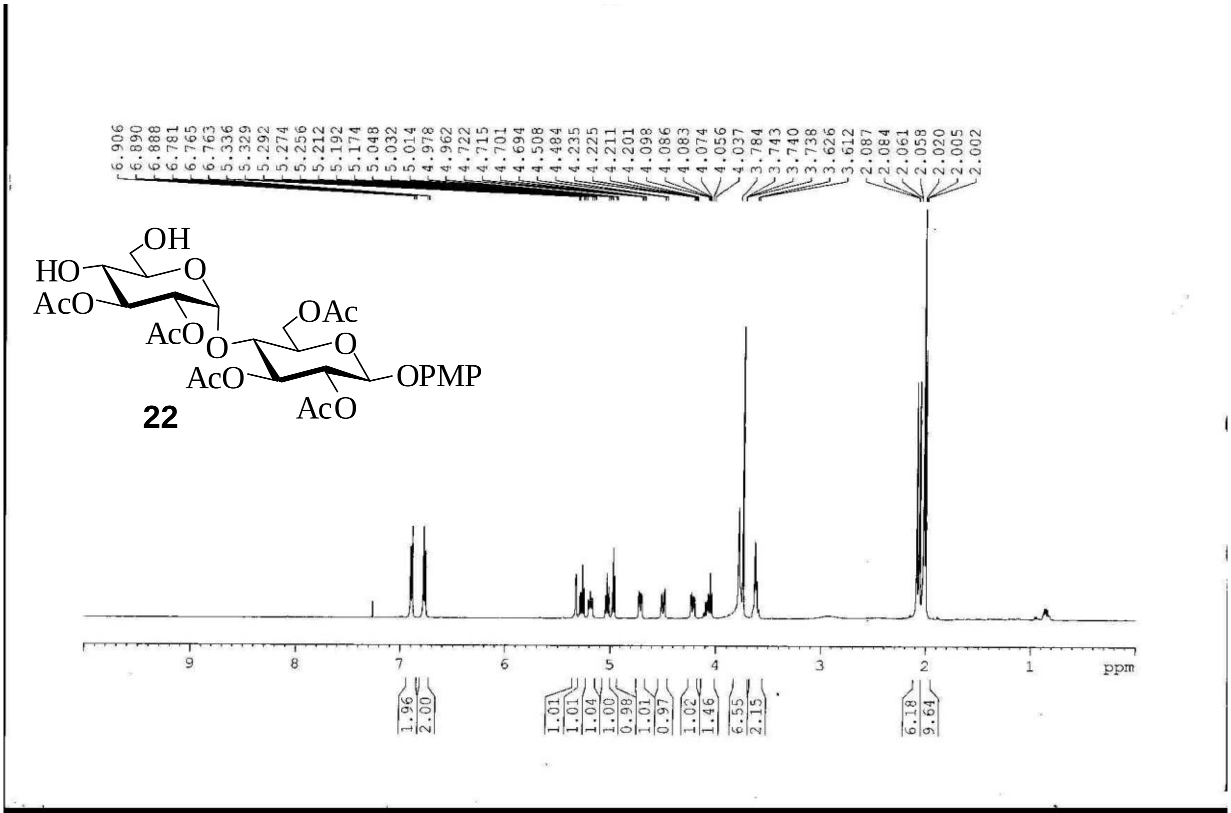

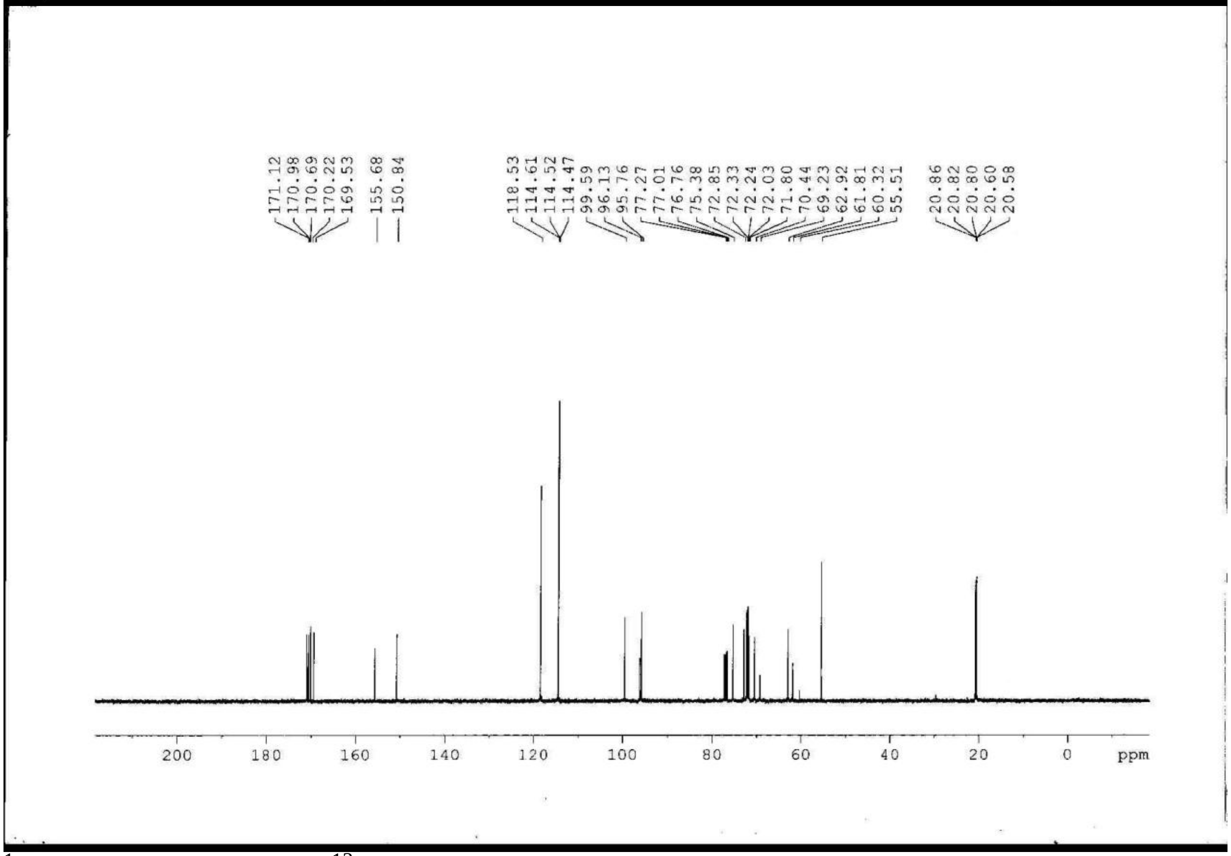

^{1}H NMR (500 MHz) and ^{13}C NMR (125 MHz) spectra in $CDCl_3$.

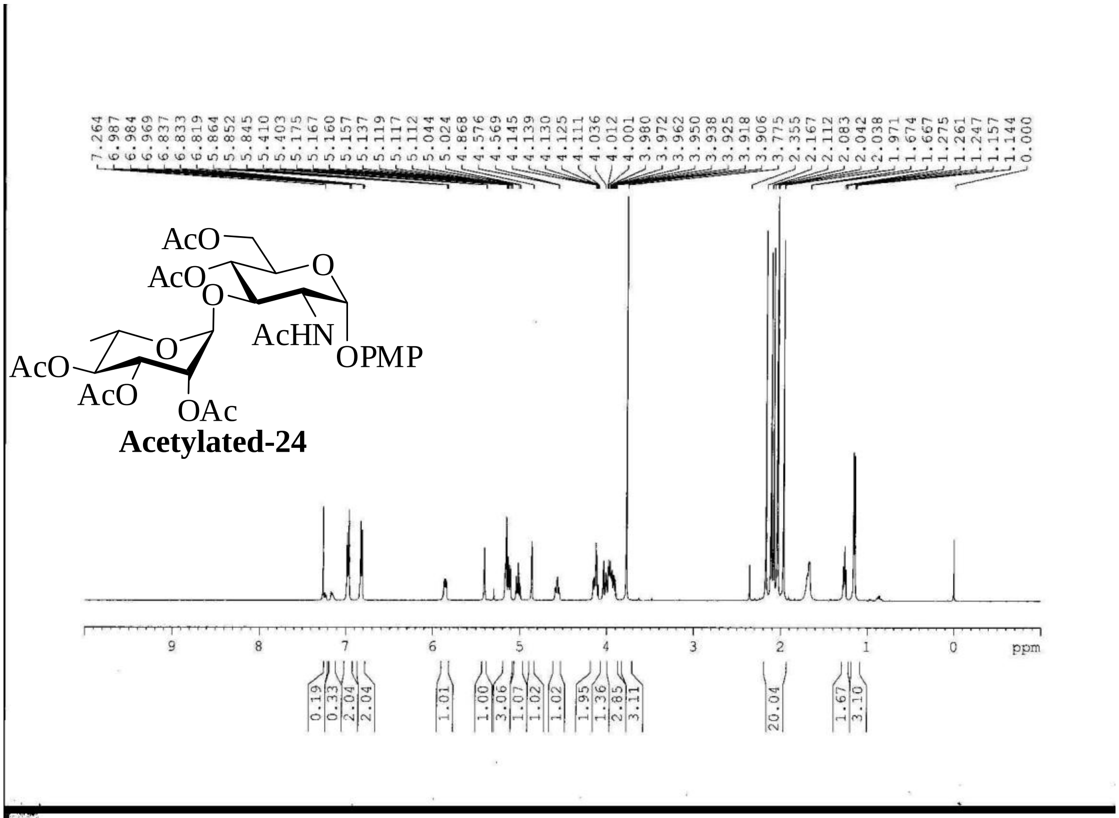

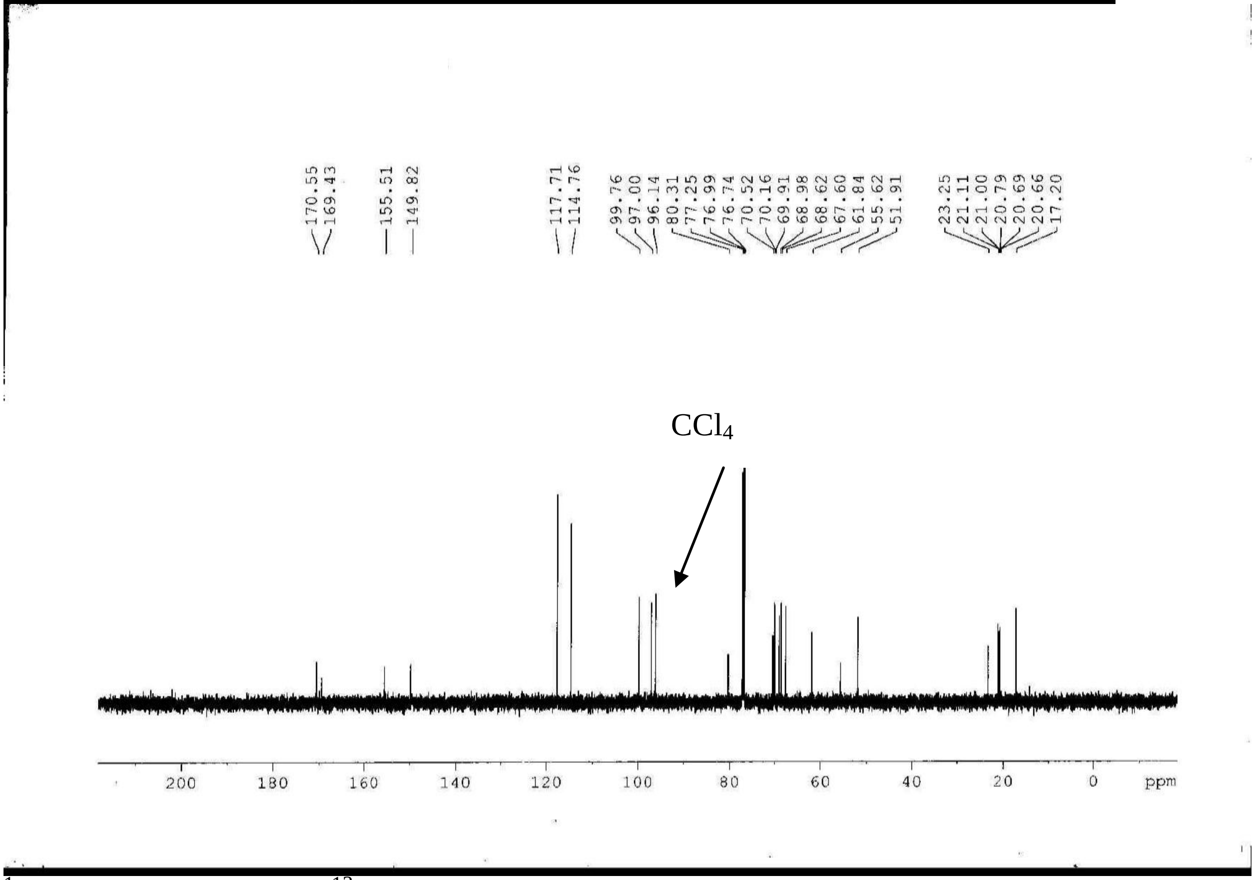

^{1}H NMR (500 MHz) and ^{13}C NMR (125 MHz) spectra in $CDCl_3$.

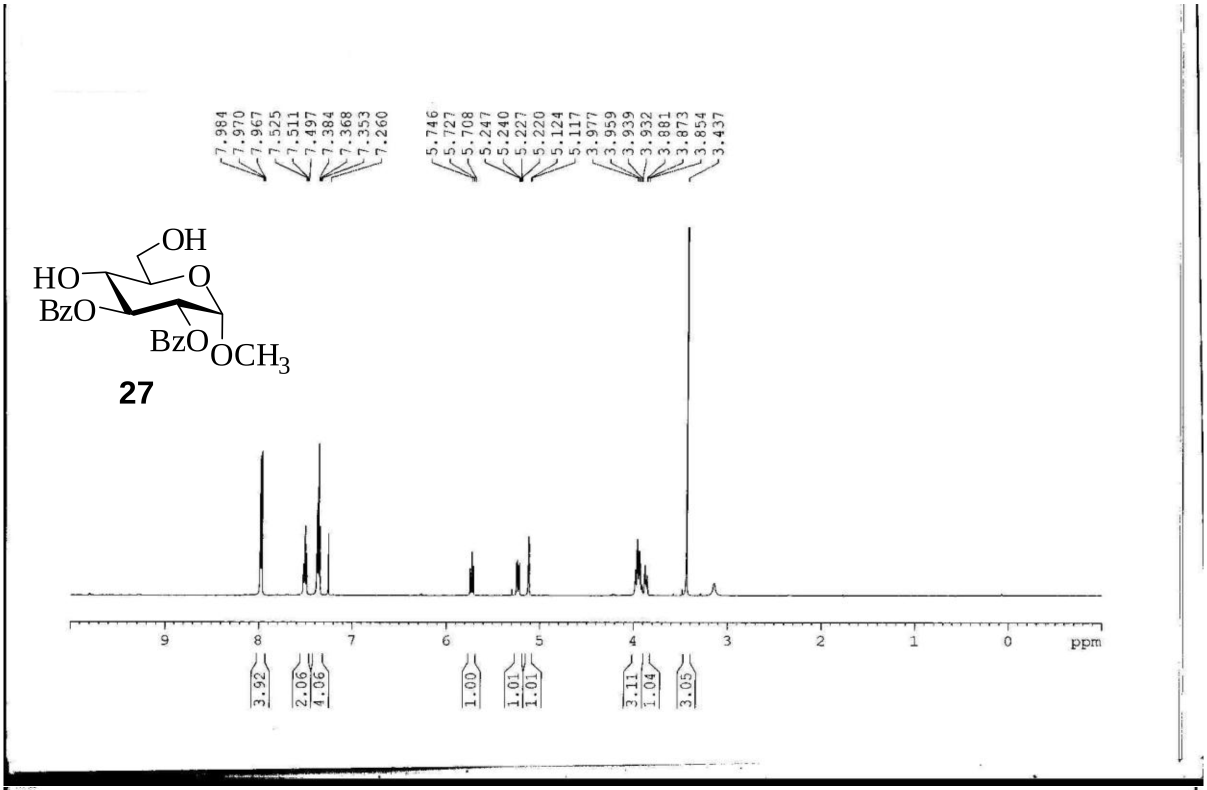

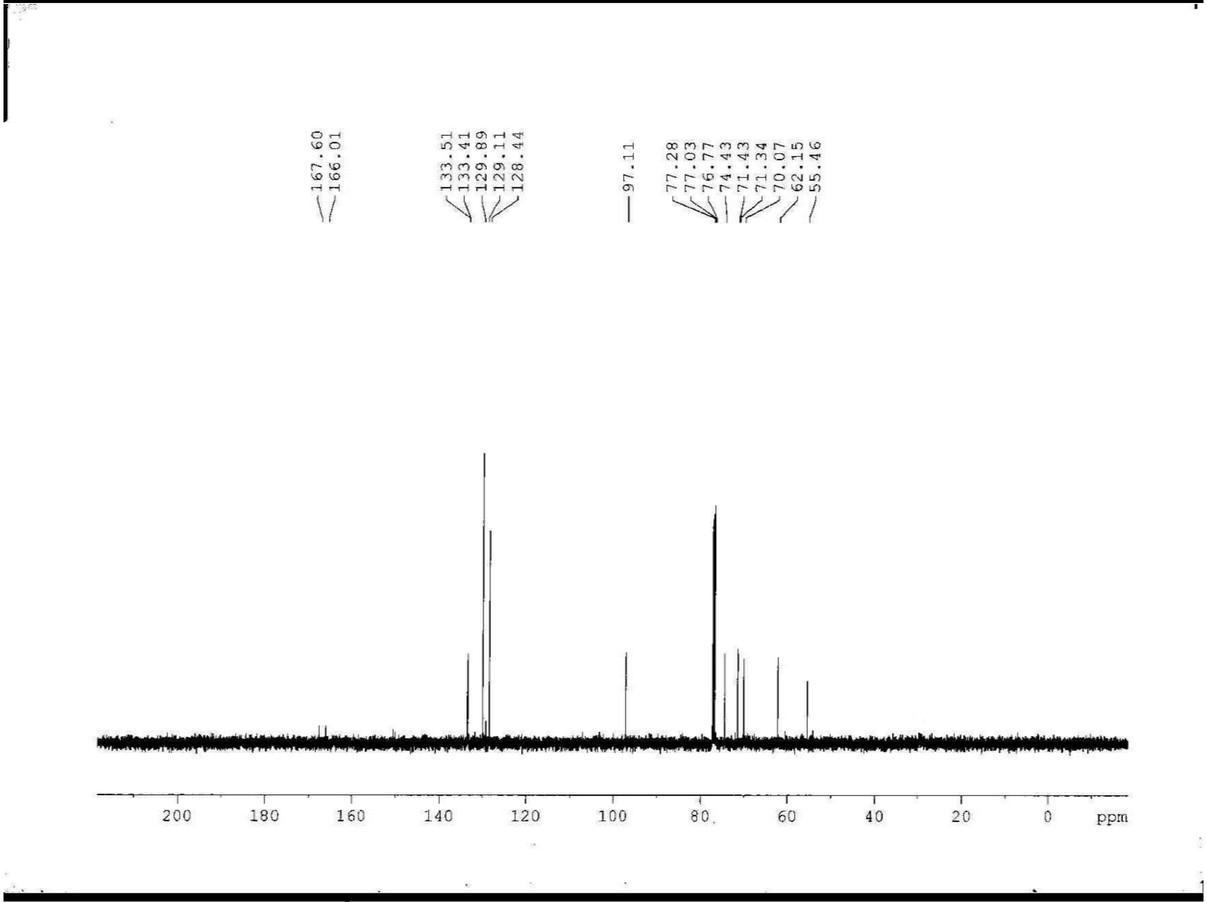

^{1}H NMR (500 MHz) and ^{13}C NMR (125 MHz) spectra in $CDCl_3$.

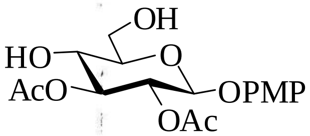

29

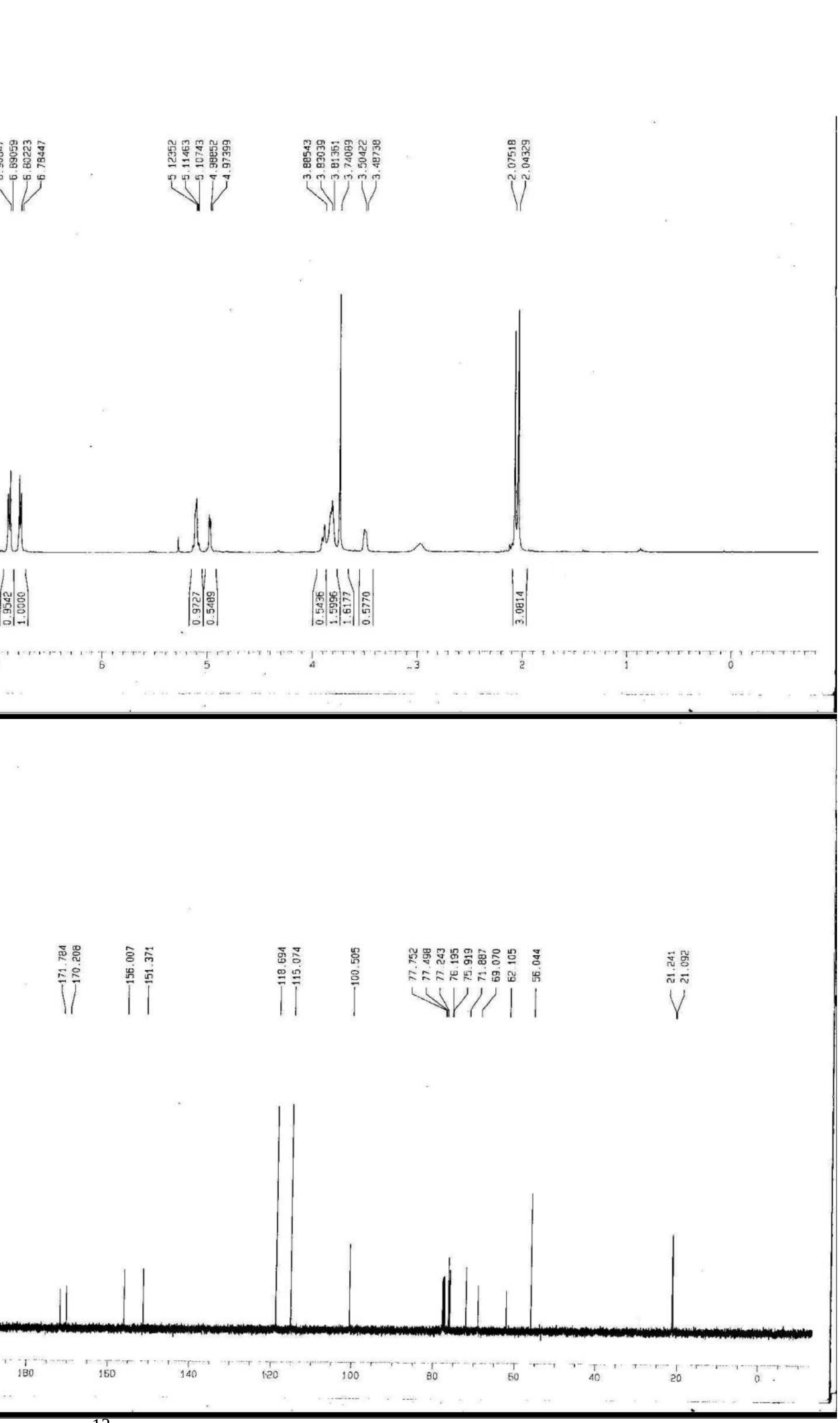

^{1}H NMR (500 MHz) and ^{13}C NMR (125 MHz) spectra in $CDCl_3$.
